# The Ethics of Traditional Chinese and Western Herbal Medicine Research: Views of Researchers and Human Ethics Committees in Australia

**DOI:** 10.1155/2011/256915

**Published:** 2010-12-13

**Authors:** Caroline A. Smith, Ros Priest, Bridget Carmady, Suzannah Bourchier, Alan Bensoussan

**Affiliations:** Centre for Complementary Medicine Research, The University of Western Sydney, Locked Bag 1797, Penrith South DC, NSW 2751, Australia

## Abstract

Despite the growth of traditional Chinese medicine (TCM) and western herbal medicine (WHM) research in Australia, little is known about how ethics committees (HRECs) assess the ethics of TCM or WHM research. The objectives of this study were to examine the experiences of TCM and WHM researchers and HRECs with the evaluation of ethics applications. Two cross-sectional surveys were undertaken of HRECs and TCM and WHM researchers in Australia. Anonymous self-completion questionnaires were administered to 224 HRECs and 117 researchers. A response confirming involvement in TCM or WHM research applications was received from 20 HRECs and 42 researchers. The most frequent ethical issues identified by HRECs related to herbal products including information gaps relating to mode of action of herbal medicines and safety when combining herbal ingredients. Researchers concurred that they were frequently requested to provide additional information on multiple aspects including safety relating to the side effects of herbs and herb-drug interactions. Overall adherence with the principles of ethical conduct was high among TCM and WHM researchers although our study did identify the need for additional information regarding assessment of risk and risk management.

## 1. Introduction

Complementary Medicine (CM) is an inclusive term that incorporates complementary medicines and complementary therapies (modalities and or systems). We use this term to include the concepts of health and medical systems, practices and products not currently recognised as part of mainstream conventional or mainstream medicine, alternative medicine (CM used in place of Western medicine), traditional medicine (indigenous medicine and practices), and integrative medicine (CM used together with mainstream Western medicine) [1]. Worldwide there is growing use of CM. The World Health Organisation estimates that 80% of the world's population depends on use of CM, including traditional indigenous medicines. Use of CM in the United States of America has been reported to be 36%, or 62% if prayer and mega vitamins are included [2]. In Australia, use of CM has increased steadily over the last twenty years. In 2006, the National Prescribing Service national consumer survey showed a significant increase in the proportion of people taking CM (67%) [3], with self-prescribed vitamins, herbal medicines, and mineral supplements the most commonly used CM. A 2006 Australian Bureau of Statistics survey reported that 3.8% of the population had consulted a CM practitioner in the previous two weeks, 0.5% had visited an acupuncturist, and 0.3% a herbalist [4]. A more recent survey of CM use in Australia indicated 46% used vitamins not prescribed by a doctor, 19% used herbal medicines, and 6% traditional Chinese medicine (TCM) [5]. 

Herbal medicines are commonly prescribed in Australia to treat a broad range of health conditions. Chinese herbal medicine, a modality that originated in China, is prescribed by practitioners qualified and trained in TCM. These practitioners are also trained to practise acupuncture. Western herbal medicine (WHM) is commonly provided by practitioners trained as Western herbalists or naturopaths and this modality, as well as the terminology, reflects the historical developments of herbal medicine through the European and American traditions. The uptake of CM in the community highlights the importance of establishing a scientific base for the safety and effectiveness of these modalities. In Australia, clinical research on TCM and WHM has mostly been restricted to academic teaching institutions delivering TCM and WHM tertiary courses, and a number of hospitals with individual researchers interested in these disciplines, as well as industry-based researchers. A survey conducted in 2007 identified 47 centres, and 253 full time researchers working in CM research in Australia [6]. Significant research activity was undertaken in the disciplines of WHM and TCM, with less research activity within other CM modalities. In late 2006, the Australian National Health and Medical Research Council (NHMRC) allocated a $5 million funding to a research grant initiative targeting CM research. This funding initiative drew a strong response demonstrating the high level of interest in CM research.

There has been limited attention given to the ethics of conducting scientific CM research and practice. In clinical practice, one qualitative study has indicated a lack of standards with respect to informed consent across a range of CM modalities [7]. Whilst commentators contend that public health and safety demand all CM adhere to the same ethical standards as for mainstream clinical research, and placebo controlled trials should be used to assess the efficacy of complementary treatments where feasible [8, 9], others propose the paradigms of CM including TCM and WHM cannot be evaluated accurately using placebo randomised controlled trials. For example, the practices of TCM and WHM can be described as complex modalities using multiple interventions with individualised treatments for each patient. These characteristics do not fit easily within the placebo control model, and are more suited to pragmatic trial designs that accommodate complex whole systems interventions. However, many TCM and WHM researchers have demonstrated an acceptance that CM can be rigorously evaluated applying randomised trial designs. Whilst TCM and WHM present researchers with challenges, for example, the standardisation of herbal medicines, drug-herb interactions, use of appropriate placebo controls and potential adverse events, the ethics of conducting TCM, and WHM research in principle should not differ from other areas of health research. 

The values of respect, research merit and integrity, justice, and beneficence, and risk safety assessment have become important ethical principles with the conduct of human research. These ethical principles theoretically should not be problematic for TCM or WHM research. However, as highlighted by Zaslawski 2008, the merit and integrity of TCM or WHM ethics applications may present challenges for researchers preparing ethics applications, and ethics committees responsible for reviewing these applications who may be unfamiliar with TCM practice principles [10]. Acupuncture research, using single blind studies and placebo controls may require engagement in active deception and limited disclosure between researchers with potential participants. This may be ethically unacceptable to some researchers and ethics committees. The risk-safety profile for acupuncture studies are low [11], however, the safety of Chinese herbs is less well established [12], although in Australia herbal products are approved as low risk products. 

In 2008, funding was received by the National Institute of Complementary Medicine (NICM) to undertake “network building” in relation to TCM. The aim of this capacity building project was to enhance research networks in TCM through creating and coordinating human resources in clinical trials, phytochemistry, and acupuncture research, and to develop methodological approaches relevant to these fields. The need to build capacity was identified in response to the growth and trends in CM research particularly in herbal medicines, and the need to establish the evidence base through use of randomised controlled trials. This initiative identified a specific need to build capacity among appropriately skilled TCM and WHM researchers, and to assist institutional ethics committees with their assessment of applications in this new field of research. Two specific areas were identified. Firstly, the complexity of TCM and WHM products with multicomponent ingredients which increases the complexity in understanding the risks and potential mechanisms. Secondly, the assessment and understanding of the low risk of products which are already available over the counter and therefore approved for human use, or they form part of the food-herb-drug interface, and the quality control and stability of herbal products. 

In light of the growth of TCM and WHM research in Australia, little is known about how HRECs assess the ethics of TCM research, or engage with TCM or WHM researchers. The objectives of this study were firstly, to examine the experiences of HRECs with the evaluation of TCM and WHM ethics applications, and secondly, to examine the experiences of TCM and WHM researchers with the submission of their research proposals to HRECs.

## 2. Subjects and Methods

### 2.1. Study Populations

Following ethics approval from The University of Western Sydney, two concurrent surveys were undertaken between May and July 2009. Firstly, all HRECs in Australia were identified from a listing held on the NHMRC database (http://www.nhmrc.gov.au/health_ethics/hrecs/hreclist.htm). A survey was sent to the HRECs and the Chair or Ethics Officer was asked to complete the survey. Secondly, a survey was sent to all identified TCM and WHM researchers in Australia. Researchers were identified from a listing of active researchers held by NICM, who had previously given permission for their contact details to be used for research purposes. In addition, new researchers were identified from the Australian and New Zealand Clinical Trial Registry (http://www.anzctr.org.au/) and a search of databases publishing in the discipline of TCM and WHM, for example PubMed (http://www.ncbi.nlm.nih.gov/). All researchers and HRECs were invited to participate. The methodological challenges of internet surveys including lower responses rates [13], and incomplete electronic addresses for all study participants, influenced our choice to administer a postal survey. Potential participants were mailed an anonymous questionnaire and covering letter explaining the purpose of the survey. A second followup mail out was made six weeks after the initial mail out. Participants were invited to return the questionnaire by email, fax, or using a reply paid envelope.

### 2.2. Questionnaire

We designed two short self-completion questionnaires (see supplmentary material available online at doi:10.1155/2011/256915). Both questionnaires included filter questions to identify responders who had submitted or reviewed a TCM or WHM ethics application in the previous three years. The questionnaire for HRECs contained 14 items. The first four items included background questions relating to the total number, and details of TCM or WHM applications reviewed, for example, modality. The next section contained items relating to composition of the HREC including number of members with a background in TCM or WHM and the evaluation methods used to assess such research applications. A further section examined the methodological and ethical complexities relating to TCM or WHM research, for example, information on herbal characterisation or standardisation, safety issues with combining herbal ingredients, or knowledge of traditional diagnostic methods. The final three questions examined the demographic characteristics of the HREC responder. The questionnaire also included an open-ended question for additional comments. 

The researchers' questionnaire consisted of 18 items. The first four items included background questions relating to the number and details of TCM or WHM ethics applications submitted. The next section related to the researchers' experience of submitting TCM or WHM applications to ethics committees. This included generic questions relating to the type of feedback received from HRECs, for example, procedures for gaining consent. Specific questions for acupuncturists and herbalists were also included, for example, request for information relating to stability, dosage, safety, and blinding. The final five questions examined the demographic characteristics of the responder. The questionnaire also included an open-ended question for additional comments.

### 2.3. Statistical Analyses

The statistical analysis was performed using Statistical Package for the Social Sciences (SPSS version 17.0). The responses from researchers and HRECs were analysed separately using descriptive statistics.

## 3. Results

### 3.1. Response from HREC

Two hundred and twenty four questionnaires were sent out to HRECs. A response was received from 148 committees (66%), of which 20 indicated they had reviewed at least one TCM or WHM application in the previous three years. Responses indicated representation across Australian States or Territories, with 8 (42%) responses from Victoria, New South Wales 5 (26%), Queensland 3 (16%), South Australia 2 (11%), and the Australian Capital Territory 1 (5%). Fifteen (79%) responders had served on an HREC for greater than three years. Membership of the HREC was represented by a researcher(s) with a health/medical/psychological or epidemiological background (94%), lawyer (84%), minister of religion (79%), nurse (73%), clinical psychologist (26%), social worker (21%), CM researcher (10%), and various other categories, for example, pharmacist, layperson, and biostatistician.

### 3.2. Review of TCM/WHM Applications by HRECs

The number of TCM and WHM applications reviewed by HRECs was a small proportion of their work. The majority of HRECs (*n* = 10, 55%) reviewed 1 to 2 applications, four committees (22%) reviewed 3 to 5 applications, and four committees had reviewed more than five (22%) applications. Eighty-five percent of HRECs reviewed applications using a randomised controlled trial (RCT). Over 50% of committees had reviewed applications on acupuncture and ingested TCM herbs, 45% reviewed ingested WHM, and a smaller number of applications relating to topically applied WHM, and therapeutic exercise, for example, Tai chi and qi gong, acupressure and massage (Table 1).

Capacity to review TCM and WHM applications was well represented with nine (45%) HRECs having members with expertise or knowledge of TCM or WHM. Six committees had two or more members with TCM or WHM background. A significant proportion of HRECs (16, 80%) were able to use the experience of committee members or subcommittees to assist with the evaluation of the scientific validity of TCM or WHM applications, or HRECs consulted with personnel within their institution (8, 40%), or referred to current literature (7, 35%). The most frequent strategy employed to address problems raised during the assessment process included seeking further clarification or information from the researcher (18, 94.4%), or requesting alteration to the study design (9, 47%). Three HRECs dismissed applications due to a lack of scientific rigour.

HRECs were asked to comment on the information provided by researchers on the methodological and ethical complexities of conducting TCM or WHM research. The aim of these questions was to identify any information gaps that may hinder the passage of applications through ethics. The responses from HRECs suggest sufficient information was provided by researchers on the blinding of studies (Table 2). Responses highlighted information gaps detailing mode of action of herbal medicines, safety when combining herbal ingredients, reliance on anecdotal evidence for phase one trials, individualisation of treatment, description of the complex intervention, and traditional diagnostic methods. HRECs requested supplementary information from researchers on data safety regarding the use of laser acupuncture, the need to explain risks to participants, risk of cross-contamination and the risk of adverse events. 

Fifty percent of HRECs had identified an ethical issue when reviewing a TCM or WHM application (Table 3). The most frequent ethical issues identified related to herbal products specifically, assessing the clinical application claims of formula, and the interaction and effect of combining herbal ingredients. Other less common ethical concerns related to the product quality of herbs, Australian regulation of the modality, and conflict of interest of the researchers due to their belief in the value of the modality conveyed in their protocol.

### 3.3. Submission of TCM and WHM Ethics Applications by Researchers

Questionnaires were sent to 117 TCM and WHM researchers, 60 questionnaires were returned (51%), of which 39 responders indicated they had submitted an ethics application as a principal investigator within the last three years. Forty-three percent of researchers responding to the survey were from New South Wales and 33% from Victoria, with smaller numbers from three other States (Table 4). Researchers were mostly male (53%), aged 35–44 years (41%), and over 64% held a Doctor of Philosophy (Ph.D.). Due to the anonymous nature of the survey, limited comparisons could be made between responders and nonresponders. Sixty eight percent of questionnaires were sent to male researchers, and a comparison with male responders suggest no difference by the sex of the responder (*P* = .09).

Fifty percent of researchers indicated they had submitted one application to an HREC in the past three years, six (16%) had submitted two applications, and eight (22%) indicated five or more submissions had been made. Over 50% of researchers had submitted an ethics application on acupuncture, fewer applications were submitted on ingested TCM and WHM herbs, and other modalities, and 80% of applications were RCT designs (Table 1). Researchers' responses suggest a greater number of acupuncture submissions were made to HRECs compared with the numbers reportedly assessed by HREC. The proportion of other TCM and WHM modalities submitted by researchers and reviewed by HRECs were similar.

Most researchers were requested to provide additional information to support their ethics application (Table 5). Two areas were frequently identified by herbal medicine researchers as requiring additional information including the side effects of herbs, and safety for herb-drug interactions. Other areas included dosage levels and interaction and cumulative effects of combined herbal ingredients. Acupuncturists were requested to provide additional information on the safety of acupuncture and potential risks, blinding, and clarification of placebo acupuncture. 

Over forty percent of researchers reported they had been requested by an HREC to amend their study protocol, this included changes to exclusion criteria, details on potential adverse events, or risks and procedures. Two responders (1.7%) indicated ethics approval had not been given to all of their TCM or WHM research. Many researchers received generic feedback requesting clarification or changes to their application. Examples included clarification or changes to language used on information sheets (47%), language used on consent forms (44%), procedures for gaining consent (36%), assessment of the study population (34%), and maintaining the scientific validity of their research (13%). Other examples included clarification regarding potential side effects, the need for indemnity insurance, role of investigators, and recruitment strategies.

The survey identified a need amongst researchers to receive further training to assist with preparation of ethics applications. Almost 80% of researchers reported their knowledge or confidence with preparing ethics applications, or responding to questions from ethics could be improved. The majority of HRECs and researchers identified an interest in receiving guidelines or further training.

## 4. Discussion

This is the first study to our knowledge reporting on the experiences of researchers and HRECs providing insight into the process of ethical submission and review of TCM and WHM research. Respondents to this survey include a small active group of clinical researchers and HRECs which reflects TCM and WHM being concentrated within a limited number of academic institutions in Australia. 

The primary purpose of the ethical guidelines for research involving humans is to promote ethically good human research. These principles of ethical conduct cover the principals of merit and integrity, justice, beneficence, respect, risk and benefit, and participants' consent [14]. Many Australian research institutions conduct a process of peer review prior to assessment by HRECs for unfunded research. Competitive research funding is usually peer reviewed and together these two institutional processes assess the research merit and integrity of the proposed TCM and WHM research, prior to assessment by the HRECs. 

Overall adherence with the principles of ethical conduct was high among TCM and WHM researchers although our study did identify some gaps mostly relating to the assessment of risk and risk management prior to approval being given. Ethics committees would normally require information presented on the toxicology, pharmacological profile and adverse events to evaluate the risk safety benefit [10]. With the development of pharmaceuticals, a step by step process of drug testing occurs in which a compound is isolated, tested in tissue cultures and animals and then investigated in phase I, II, and III trials in humans. Although Chinese herbs have been used for many centuries the safety profile is not well established [12] and it is rare for a strong preclinical basis for dosing to exist, and reliance is made on anecdotal evidence. Consequently, further information may be requested by HRECs on the characterisation, toxicity, and active constituents as demonstrated by our findings. CONSORT (Consolidated Standards of Reporting Trials), a statement describing the reporting of herbal interventions, serves to raise awareness to the importance of reporting this information to stakeholders involved in all phases of the research process [15], and Tilburt and Kaptchuk 2008 suggest many researchers already recognise the need to establish a rational basis for dosing and standardisation of biological active compounds before initiating large treatment trials [16]. 

It was encouraging to find the majority of researchers did not need to make significant amendments to their study protocols, however, among those reporting a need to make changes risk and risk management were recurring themes. HRECs reported insufficient information was presented on safety when combining herbal ingredients, claim of formula, drug-herb interactions, and product quality of herbs. This mirrored feedback from researchers indicating they were requested to provide additional information on the characterisation, stability, and standardisation of herbal products safety and risks from acupuncture. Overall adherence with other ethical principles was high and less problematic. HRECs identified 20% of research applications received having a conflict of interest relating to financial interests or personal benefit which may lead to the research process being compromised. 

Lack of disclosure to research participants about the use of placebo acupuncture has been reported in four RCTs published in leading medical journals [17], and a review of acupuncture placebo controlled trials [18]. Trials where participants were told they would be randomly assigned to different forms of acupuncture rather than a placebo control have been described as “deceptive disclosure”. This was not an area of acupuncture research identified as having an ethical concern in our study.

There has been a perception that some ethics committees may be biased against CM research [9], however, the findings from our study do not support this view. The majority of HRECs appeared to have access to expertise (although the criteria of expertise remains unclear) to review TCM or WHM applications when the need arose. Whilst our findings provide very few cases of research being dismissed due to scientific rigour, there is no data to ascertain if these applications were unfairly judged. 

Researchers and members of HRECs expressed an interest in receiving further training. Two initiatives are underway which will provide researchers and HRECs with useful resources. Standard operating procedures (SOPs) relating to TCM research and publication of frequently asked questions are in the final stages of development at The University of Western Sydney, and these will be made widely available in Australian and international TCM networks. Standard operating procedures (SOPs) will develop and improve operating procedures and will allow for certification or an accreditation programme in the future. 

The limitations of our study primarily relate to the response and participation rates. It is not known precisely how many researchers have undertaken TCM or WHM research in Australia. We were only able to target those researchers for which we had contact details. Consequently it is not possible to determine what proportion of researchers the survey represents. A 50% response rate from researchers suggests our findings may not be generalisable to all TCM and WHM researchers. Selection bias must be considered, and it is possible that only the most motivated researchers responded to the survey. We have limited data available on the non responder and it is unclear if our results reflect the experience of early or mid career researchers. However, one comparison of the sex of responder and the study population suggests responders were representative of TCM and WHM researchers. In addition, responses from researchers mirrored the geographical distribution and the ethics review activity of HRECs. We consider the geographical distribution of responding HRECs to be representative of HRECs reviewing TCM and WHM ethics applications in Australia.

Future research or replication of this study in other geographical settings would be assisted by a census of researchers active in respective disciplines. In addition, the content validity of the questionnaire could be established from piloting of the questionnaire in different geographical settings and with similar CM disciplines. We were unable to validate responses from participants in our study due to the lack of followup, and there was limited opportunity for in-depth questioning relating to the type or extent of TCM and WHM knowledge of HREC members. These areas could be explored further using qualitative methods.

## 5. Conclusion

This study has shown that TCM and WHM researchers and HRECs in Australia have valuable experience with the submission and review of applications, and the majority of researchers successfully negotiate the ethics processes. Overall adherence with the principles of ethical conduct was high among TCM and WHM researchers although our study did identify some gaps mostly relating to the assessment of risk and risk management.

##  Disclosure

The authors have no potential conflict of interest to disclose.

## Figures and Tables

**Table 1:** Modality of traditional Chinese medicine (TCM) or Western herbal medicine (WHM) reviewed by HRECs or submitted by a researcher.

Modality of TCM or WHM submitted	Reviewed by Ethics		Submitted by researcher	
	*n* = 20	%	*n* = 39	%
Acupuncture	10	50.0	20	51.3
Ingested TCM herbs	10	50.0	13	33.3
Ingested WHM herbs	9	45.0	8	20.5
WHM topically applied	3	15.0	1	2.6
Therapeutic exercise	4	20.0	3	7.7
Acupressure	1	5.0	2	5.1
Massage	2	10.0	1	2.6

Number (*n*) and percentage (%).

**Table 2:** Information on the methodology of the ethics applications assessed by HREC as sufficient.

Domain	*n* = 20	%
*Blinding or lack thereof*	16	80
*Mode of action from a scientific perspective*	10	50
*Safety issues when combining herbal ingredients*	8	40
*Limited phase one and reliance on anecdotal evidence for safety and dosage *	6	30
*Individualisation of treatment according to diagnosis*	5	25
*Herbal characterisation, standardisation, and stability*	5	25
*Knowledge of traditional diagnostic methods*	4	20
*Description of a complex intervention or multi modality holistic treatment*	4	20

Number (*n*) and percentage (%).

**Table 3:** Ethical issues encountered by HREC with reviewing TCM or WHM applications.

Ethical issue	Yes	
	*n* = 20	%
Assessment of clinical application claim of formula	10	50.0
Interaction and effects of combining herbal ingredients	10	50.0
Product quality regarding herbal interventions	7	35.0
Information regarding Australian regulation of modality	7	35.0
Conflict of interest for research due to belief in value of their modality	6	30.0
Conflict of interest for researchers regarding transparency of funding	4	20.0
Surveys creating unnecessary anxiety, or raised expectations	2	10.0

Number (*n*) and percentage (%).

**Table 4:** Characteristics of researchers responding to the survey.

Demographic	Researcher	
	*n* = 39	%
Response by State		
Victoria	13	33.3
New South Wales	17	43.6
Queensland	6	15.4
South Australia	2	5.1
Western Australia	1	2.6
Age (years)		
18–34	5	12.9
35–44	16	41.0
45–54	11	28.2
55–64	7	17.9
Highest qualification		
PhD	25	64.1
Masters	4	10.3
Bachelors with Honours	4	10.3
Bachelors/Graduate Diploma	6	15.4

Number (*n*) and percentage (%).

**Table 5:** Requests for further information to support researchers ethics applications.

Chinese herbal or Western herbal medicine researchers	Yes	
	*N* = 24	%
Safety for herb-drug interaction	12	50.0
Side effects of herbs	12	50.0
Dosage levels	8	32.0
Interaction and cumulative effects of combined herbs when using formulas	7	28.0
Characterisation to quantify chemical constituents	5	20.8
Standardisation of therapeutic active compound	4	16.0
Stability and shelf life	2	8.0

Acupuncture researchers	*N* = 22	%

Safety of acupuncture and potential risks	9	40.9
Assessment of blinding	7	31.8
Clarification of sham acupuncture	6	27.3
Action of specific acupuncture points	2	9.1
Clarification of term differential diagnosis	2	9.1

Number (*n*) and percentage (%).
